# Ultrasound-Guided Transthoracic Mediastinal Biopsy: A Safe Technique for Tissue Diagnosis in Middle- and Low-Income Countries

**DOI:** 10.7759/cureus.13914

**Published:** 2021-03-16

**Authors:** Muhammad Kashif Shazlee, Muhammad Ali, Muhammad Saad Ahmed, Junaid Iqbal, Jaideep Darira, Muhammad Qasim Naeem

**Affiliations:** 1 Diagnostic Radiology, Dr. Ziauddin Hospital, Karachi, PAK

**Keywords:** ultrasound guided transthoracic mediastinal biopsy, mediastinal mass, mediastinal tumours, mediastinal biopsy associated mortality, mediastinal biopsy related complications

## Abstract

Background and objectives

The high cost of video-assisted transthoracic procedures precludes their use in the diagnostics of mediastinal masses in low- and middle-income countries (LMICs). This study aims to assess the technical success rate and diagnostic yield of ultrasound-guided transthoracic mediastinal biopsies at a tertiary care hospital.

Methods

This descriptive cross-sectional study was conducted in patients presenting with mediastinal masses referred to radiology services at Dr. Ziauddin University Hospital. Karachi, Pakistan. Ultrasonography was performed using Toshiba Xario 200 & Aplio 500 using convex and linear probes accordingly. Biopsy was performed using a combination of 18G semiautomatic trucut and 17G co-axial needles. Complications and overall diagnostic yields were determined.

Results

In all 70 patients referred, the procedure was completed successfully with an overall procedural yield of 95.7%. Inconclusive biopsies due to inadequate specimen were seen in two (4.2%) patients. No post-procedure major complication or mortality was observed. Minor complications were seen in three (4.2%) out of 70, including hematoma (<3 cm) in one patient and small pneumomediastinum in two patients.

Conclusion

Ultrasound-guided transthoracic mediastinal biopsy may be the pragmatic technique of choice in LMICs for the diagnosis of mediastinal masses as they provide real-time visualization and is cost-effective and safe.

## Introduction

Anterior mediastinal masses could be a sign of large-spectrum underlying pathologies ranging from benign masses to malignant tumors [1]. Accurate and early diagnosis in this regard can be that fine line that distinguishes complete cure and recovery from mortality. For instance, timely diagnosis of thymic malignancy of advanced stage can be successfully treated with neoadjuvant treatment before surgery [2]. Similarly, lymphomas and germ cell tumors, among other malignancies, can be treated if early diagnosis is made [3-6].

There are numerous other examples that show the importance of early diagnosis and the potential benefits gained from it [7,8]. However, gold standard histopathology-based diagnosis needs tissue sample for which the access to the mediastinal mass is required. There are several methods for obtaining tissue samples for the diagnosis of mediastinal lesions. These include surgical techniques such as video-assisted thoracoscopic biopsy, transbronchial needle biopsy, and endoscopic ultrasound (US)-guided fine-needle aspiration biopsy.

In order to achieve optimal access to the mass, the video-assisted transthoracic surgery (VATS) is deemed superior to other techniques due to adequate visualization despite limited access to the mediastinum. However, VATS cost approximately 1,400 USD per procedure, making this technique unfavorable in a resource-limited country like Pakistan. Therefore, accurate, timely, and cost-effective diagnosis of such masses is of crucial importance.

In Pakistan, the double burden of communicable and non-communicable diseases has posed an even greater risk to the misdiagnosis, or worse, no diagnosis at all, henceforth grave clinical outcomes in the situation where high-fidelity diagnostic techniques are unaffordable or unavailable to the patients. Our study aims to evaluate the technical outcomes of US-guided transthoracic mediastinal biopsies in patients presenting with mediastinal masses for tissue sampling for histopathology at a tertiary care hospital.

## Materials and methods

This prospective study was conducted between October 2013 and October 2016 and included 70 consecutively enrolled patients referred to the department of radiology, Dr. Ziauddin University Hospital, Karachi, Pakistan, for histopathological tissue sampling of mediastinal masses or insinuating mediastinal lesions with anterior or posterior compartment involvement, as seen on cross-sectional contrast imaging followed by US-guided transthoracic mediastinal biopsy. The study was conducted following prior approval from the Institutional Ethical Review Committee.

After reviewing the scans, only those patients with adequate acoustic window of the mass were included. Patients with pure cystic lesions or widely necrotic tissue, INR greater than 1.8, inability to maintain the desired position for the procedure, and pediatric patients with any contraindication to general anesthesia were excluded.

In pediatric patients, the procedure was performed under general anesthesia, whereas local anesthesia was used in adults. Ultrasonography was performed using Toshiba Xario 200 & Aplio 500 (Toshiba Medical Systems, Tokyo, Japan) using convex and linear probes accordingly. Color Doppler imaging was used in all patients to assess the location of large vessels in close proximity. Co-axial US-guided transthoracic mediastinal biopsy was performed with the needle directed away from the major vessels and heart with a maximum three passes in each patient. All biopsies were taken with a combination of 18G semiautomatic trucut needle and 17G co-axial needle. In lesions with necrosis, peripheral soft tissues (enhancing on contrast CT) were targeted. If the parasternal approach was not possible, transsternal (in the pediatric group), suprasternal, or paravertebral approaches were employed.

All patients were kept under observation for the next four hours for monitoring of vitals as per department protocol. A follow-up erect chest radiograph was obtained after one hour in all patients. US Doppler evaluation was repeated prior to discharge. Histological samples were stored in formaldehyde solution and sent for histopathological reporting. These were collected for final diagnoses.

## Results

The mean age of the patients was 32.45±31 years (range: 2.5 years to 70 years). Of all the patients, 58 (82.8%) were males and 38 (53.2%) were pediatric patients.

The majority of cases had masses within the anterior mediastinum (42.8% [n=30]) followed by the middle mediastinum (35.7% [n=25]) and posterior mediastinum (14.2% [n=10]), whereas five (7.1%) out of 70 patients had mass in the anterior chest wall with mediastinal invasion.

The procedure was successfully completed in all patients (100% technical success rate). In 68 out of 70 patients, the tissue sample was adequate for diagnostic biopsies, giving an overall procedural yield of 95.7%. An inconclusive biopsy was reported in two (4.2%) patients due to inadequate tissue specimen, who were then suggested surgical biopsy. No major complication or mortality was observed in the study participants. Minor complications were seen in 3 (4.2%) out of 70 patients. This included a small hematoma of less than 3 cm in one patient and small pneumomediastinum in two patients. All minor complications were self-limiting as there were no clinical signs of distress or change in hemodynamics. However, a repeat radiograph was performed at the time of discharge again to confirm any progression.

Malignant masses were seen in 84.2% (n=59/70) on histopathology. Lymphoma was the most common malignancy and seen in 55.9% (n=33/59) of the patients. The most common non-malignant lesion was neurogenic tumors and seen in 63.6 % (n=7/11) of the patients.

**Figure 1:**
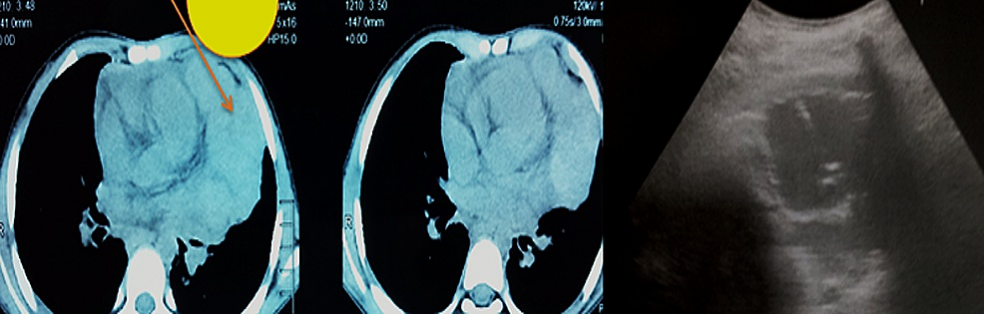
Plain CT scan of the chest showing a large anterior mediastinal mass along the pericardium. Ultrasound-guided accurate needle placement was performed for transthoracic mass biopsy.

**Figure 2:**
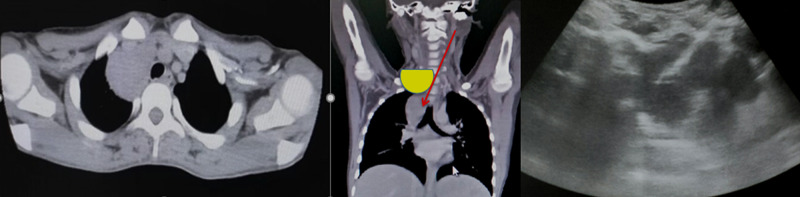
Plain CT scan of the chest showing a large anterior mediastinal mass in the right paratracheal location. Ultrasound-guided para-sternal needle placement was performed for transthoracic mass biopsy.

**Figure 3:**
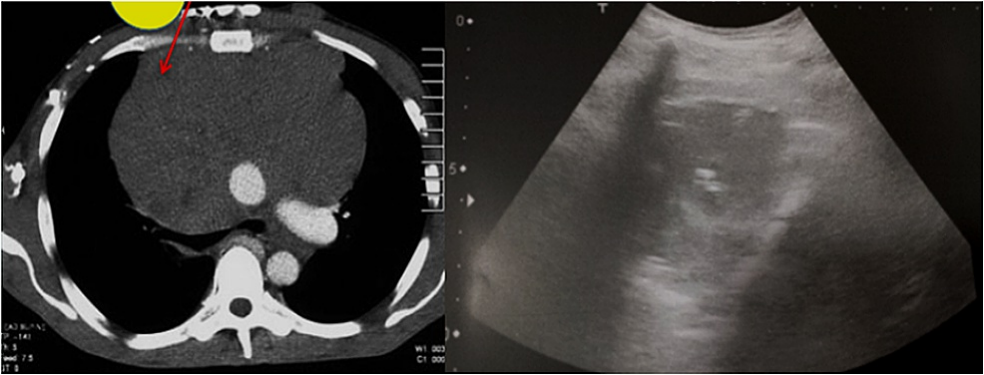
Plain CT scan of the chest showing a large anterior mediastinal mass. Ultrasound-guided para-sternal needle placement was performed for transthoracic mass biopsy.

Figures 1-3 show a large anterior mediastinal mass. US-guided parasternal needle placement was performed for transthoracic mass biopsy.

## Discussion

In the recent past, attention has been redirected towards the use of US-guided interventional procedures in terms of cost, enabling real-time visualization and repeatability without radiation hazards. In case of transthoracic mediastinal biopsy, US-guided procedures have proven to be of high accuracy, providing adequate samples for histopathology, without severe complications or highly cost [9]. Cost is of special consideration when it comes to the diagnosis of mediastinal masses in LMICs like Pakistan where the incidence of conditions leading to such masses is on the rise [10]. In our study, the average cost of the procedure was 13,000 PKR (112 USD) at our center compared to approximately 200,000 PKR (1,400 USD) for VATS. Our study findings showed excellent technical success of the procedure comparable to that of VATS.

Primary mediastinal masses account for 3% of tumors involving the chest wall [11,12]. Around 25-40% are potentially malignant [11] and are more likely to be located in the anterior mediastinum (Abstract: Eames R, Kim Y, How E, Wong L. Anterior mediastinal mass. 2019). The most common lesions encountered in the mediastinum are thymoma, neurogenic tumors, and benign cysts, altogether representing 60% of patients with mediastinal masses [12]. Correct diagnosis of a mediastinal tumor depends on clinical and radiological history, imaging findings, and histopathological diagnosis.

Of the surgical approaches, VATS is popular because of safety and accuracy as compared to cervical mediastinoscopy [13-15]. Despite this notion, the VATS is expensive procedure for middle-income country like Pakistan and even more impractical for the low-income countries. However, imaging-guided biopsy of anterior mediastinal masses offers less complications as it is less invasiveness [16-18].

## Conclusions

US-guided transthoracic mediastinal biopsy is a pragmatic technique of choice in LMICs for the diagnosis of mediastinal masses as it is cost-effective and safe, and provides real-time visualization.
